# National Prevalence and Risk Factors of Hepatitis B Virus Infection in Tunisia Two Decades After Vaccine Introduction

**DOI:** 10.3390/vaccines14050373

**Published:** 2026-04-23

**Authors:** Ahlem Fourati, Meriem Ben Hadj, Sonia Dhaouadi, Aicha Hechaichi, Hejer Letaief, Mouna Safer, Amal Cherif, Farah Saffar, Souhir Chelly, Hind Bouguerra, Asma Bahrini, Khouloud Talmoudi, Takoua Chouki, Olfa Hazgui, Naila Hannachi, Olfa Bahri, Nissaf Bouafif é p Ben Alaya

**Affiliations:** 1 National Observatory of New and Emerging Diseases, Tunis 1002, Tunisia; meriem-benhadj@hotmail.com (M.B.H.); sonidhaouadi88@gmail.com (S.D.); aicha.hechaichi@gmail.com (A.H.); hejerletaief@gmail.com (H.L.); safermouna@gmail.com (M.S.); amalcherif206@gmail.com (A.C.); farahsaffar27@gmail.com (F.S.); souhirch@hotmail.com (S.C.); hind_296@hotmail.com (H.B.); bahrini.asma@gmail.com (A.B.); talmoudi.khouloud@gmail.com (K.T.); takwachouki@gmail.com (T.C.); nissafba@yahoo.fr (N.B.é.p.B.A.); 2 Laboratory of Microbiology, Farhat Hachad Hospital, Sousse 4031, Tunisia; olfahazgui1@gmail.com (O.H.); nailahannachibensayah@gmail.com (N.H.); 3 Laboratory of Microbiology and Virology, Aziza Othmana Hospital, Tunis 1008, Tunisia; olfa.bahri@fmt.utm.tn; 4 Faculty of Medicine of Tunis, University of Tunis El Manar, Tunis 1007, Tunisia

**Keywords:** viral hepatitis B, prevalence, associated factors, seroepidemiologic studies

## Abstract

**Background/Objectives**: Tunisia lacks recent national data on hepatitis B virus (HBV) prevalence, particularly following the introduction of universal HBV vaccination in 1995. A national HBV seroprevalence study is essential to guide prevention strategies. This study aimed to estimate the national seroprevalence of HBV infection and identify its determinants 20 years after vaccine introduction. **Methods**: We conducted a nationwide, household-based, cross-sectional sero-epidemiological survey among a representative sample of the Tunisian general population using a two-stage cluster sampling method. The study was conducted by the National Observatory of New and Emerging Diseases (ONMNE) between December 2014 and June 2015. Data were collected using standardized questionnaires, and blood samples were tested using electrochemiluminescence (ECLIA) to detect HBV biomarkers (HBsAg, anti-HBc, anti-HBs). HBV infection was defined as the presence of HBsAg and/or anti-HBc with the absence of anti-HBs. Associations between HBV infection and explanatory variables (socio-demographics, vaccination status, intrafamilial transmission, and hospital exposures) were assessed using multivariate logistic regression, reporting adjusted odds ratios (aORs) with 95% confidence intervals (CIs). **Results**: Among 21,720 participants, 19,155 (88.2%) were tested. The national prevalence of HBsAg was 1.7% (95% CI: 1.55–1.85%), higher among males (2.1%; 95% CI: 1.9–2.4%) than females (1.4%; 95% CI: 1.3–1.6%) (*p* < 0.001; M/F ratio = 1.48). The mean age of HBsAg-positive participants was 48 ± 15.7 years. Prevalence was highest in the Central (2.3%; 95% CI: 2.0–2.7%) and Southern regions (2.2%; 95% CI: 1.8–2.8%) (*p* < 0.001). In multivariate analysis, independent risk factors for HBV infection included age >20 years (aOR = 15.10; 95% CI: 4.79–47.64; *p* < 0.001), having a family member with HBV infection (aOR = 2.82; 95% CI: 2.09–3.79; *p* < 0.001), residing in the Southern (aOR = 2.51; 95% CI: 1.76–2.71; *p* < 0.001) or Central region (aOR = 2.18; 95% CI: 1.76–2.71; *p* < 0.001), male gender (aOR = 1.69; 95% CI: 1.39–2.05; *p* < 0.001), and hospital follow-up (aOR = 1.23; 95% CI: 1.01–1.51; *p* = 0.039). HBV vaccination was strongly protective (aOR = 0.36; 95% CI: 0.20–0.62; *p* < 0.001). **Conclusions**: The national HBsAg seroprevalence in Tunisia was 1.7%, reflecting a low-endemic status. Vaccination programs should prioritize high-risk groups, including males, adults over 20 years, household contacts of HBV carriers, and residents of the Central and Southern regions. Strengthening infection prevention and control in healthcare settings and adopting intrafamilial precautions among high-risk populations are essential for long-term HBV control.

## 1. Introduction

Hepatitis B virus (HBV) infection remains a major global public health problem. In 2019, the World Health Organization (WHO) estimated that 296 million people were living with chronic HBV infection, with approximately 1.5 million new infections occurring each year [1].

HBV infection increases the risk of developing hepatocellular carcinoma (HCC) and cirrhosis, leading to nearly one million deaths annually [1]. Globally, in 2019, HBV-related diseases were responsible for an estimated 555,000 deaths (95% CI: 487,000–630,000), accounting for 48.8% (95% CI: 44.6–52.7%) of all hepatitis-related deaths [2]. Hepatitis B was the leading cause of liver cancer deaths, contributing to 39.5% (95% CI: 35.2–44.4), and the third leading cause of cirrhosis-related deaths, accounting for 22.5% (95% CI: 19.3–26.0) [2]. Specifically, HBV-related cirrhosis caused 331,000 deaths (95% CI: 279,000–392,000), liver cancer accounted for 192,000 deaths (95% CI: 162,000–224,000), and acute hepatitis B was responsible for 32,500 deaths (95% CI: 23,900–44,700) worldwide [2].

The risk of progression to chronic infection is strongly influenced by the age at which infection occurs. The younger the individual is at the time of HBV infection, the greater the likelihood is of developing chronic disease. Nearly 90% of infants infected with HBV will develop lifelong chronic infection, whereas this risk decreases progressively with increasing age [3].

In Tunisia, the epidemiological context of HBV infection is marked by the lack of recent national data on its prevalence. The most recent available data date back to the 1990s, when studies reported HBV infection prevalence rates ranging from 3.7% to 4.8% [4]. Based on these estimates, Tunisia was classified as a country of intermediate endemicity [5].

HBV infection has been a mandatory notifiable disease in Tunisia since 1992 [6]. Regarding prevention strategies, screening for HBV infection among blood donors was introduced during the 1970s and 1980s, and systematic screening of pregnant women has been implemented since 1996 [7,8].

Hepatitis B vaccination was introduced into the national immunization program in 1995, initially administered systematically to infants from the age of three months. Since September 2006, the vaccine has been given at birth. Vaccination has also been extended to high-risk groups, including healthcare workers since 1992 and medical and paramedical students since 2001 [9].

In 1996, two national surveys were conducted to assess the prevalence of HBV serological markers, chronic HBsAg carriage, and modes of transmission. One survey was population-based and included all age groups, while the second was conducted in schools and targeted children aged 6, 12, and 18 years (born in 1990, 1984, and 1978, respectively), who were not eligible for vaccination at that time [4,10].

Given the time elapsed since these surveys and the implementation of universal vaccination, an updated national HBV seroprevalence study is essential.

In this context, the Ministry of Health initiated a national household-based cross-sectional sero-epidemiological survey to assess the prevalence of viral hepatitis A, B, and C in Tunisia. The survey was conducted by the National Observatory of New and Emerging Diseases (ONMNE).

The primary objective of this study was to estimate the national seroprevalence of HBV infection in Tunisia and to identify associated risk factors in order to inform prevention strategies and public health recommendations. The secondary objective was to assess the effectiveness of HBV vaccination.

## 2. Materials and Methods

### 2.1. Study Design, Setting and Period

We conducted a national household-based cross-sectional sero-epidemiological survey among a representative sample of the Tunisian general population. The study was designed to estimate the seroprevalence of viral hepatitis A, B, and C and to assess associated risk factors.

The survey was led by the National Observatory of New and Emerging Diseases (ONMNE), Ministry of Health, and was carried out between December 2014 and June 2015.

Tunisia is a North African country with approximately 11 million inhabitants at the time of the survey, administratively divided into 24 governorates and characterized by diverse geographic and socio-demographic profiles (urban and rural areas). To ensure national representativeness, the survey covered all regions of the country and included participants from both urban and rural settings.

### 2.2. Study Population

#### 2.2.1. Inclusion Criteria

All household members present at the time of the investigators’ visit were eligible for inclusion, regardless of age or sex, provided that they agreed to participate and provided written informed consent. Participation required completion of the questionnaire and provision of a blood sample for serological testing.

Individuals who were absent during the initial visit were contacted and offered a second appointment. A follow-up visit was conducted within the same month to ensure maximum participation.

#### 2.2.2. Exclusion Criteria

Individuals were excluded if they were non-permanent residents of the selected district, had a medical contraindication to blood sampling, refused to provide a blood sample, or did not provide written informed consent.

### 2.3. Sampling

#### 2.3.1. Sample Size Calculation

The required sample size for the general population was calculated using Epi Info 7 software according to the following formula:

$$n=DE\times \frac{Z_{\alpha /2}^{2}\times p(1-p)}{d^{2}}$$

where

- DE is the design effect (correction factor for cluster sampling), set at 1.5;
- Z_α/2_ = 1.96 for a two-sided α of 0.05;
- p is the expected seroprevalence of HCV infection;
- d is the desired precision.

As this study was part of a national survey assessing three types of viral hepatitis (A, B, and C), the sample size was determined based on the estimated seroprevalence of hepatitis C virus (HCV) infection, which had the lowest expected prevalence. This conservative approach ensured adequate statistical power for all hepatitis types included in the survey, as recommended by the survey scientific committee [11].

To account for an anticipated 15% rate of serum loss and/or refusal to participate, the final required sample size was increased to 22,275 individuals. Assuming an average household size of four members, the estimated number of households (clusters) required was 5500 (Table 1).

#### 2.3.2. Sampling Procedure (Multistage Cluster Sampling)

The source population for this study comprised the entire Tunisian population, regardless of age, sex, or region of residence, based on the 2014 national census (N = 11,803,588). Given the logistical challenges of obtaining a complete survey frame for all individuals, a multistage cluster sampling approach was implemented to ensure a representative and feasible selection of participants across the country.

Stage 1: Selection of districts (clusters)

All 24 governorates of Tunisia were included in the sampling frame. Within each governorate, districts (census enumeration areas) were randomly selected using probability proportional to size (PPS) sampling, based on population data from the national census. This approach ensured that districts with larger populations had a higher probability of selection, maintaining representativeness at the national level.

Stage 2: Selection of households within districts

Within each selected district, households were randomly selected. A household was defined as a group of individuals living together and sharing common meals. The number of households per district was determined to meet the required total sample size, assuming an average household size of four members.

Stage 3: Selection of individuals within households

**Figure 1:**
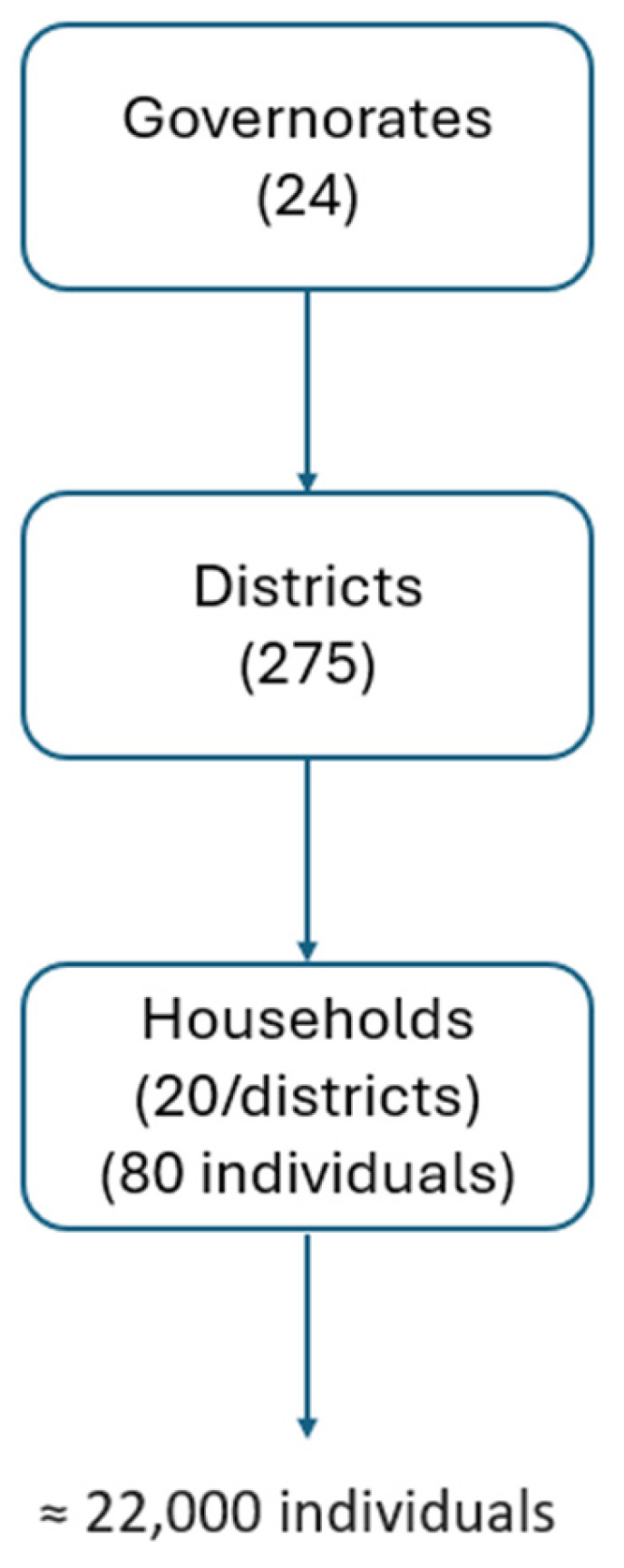
Sampling procedure; seroprevalence survey of hepatitis B infection in Tunisia, 2014–2015.

All household members present at the time of the visit and meeting the inclusion criteria were invited to participate in the survey. Individuals absent during the first visit were revisited within the same month to minimize non-response. All eligible individuals in selected households were included in the survey (Figure 1).

This multistage cluster sampling design allowed the survey to achieve adequate geographic coverage, capture both urban and rural population distributions, and ensure that the sample was representative of the overall Tunisian population in terms of age, sex, and region.

### 2.4. Operational Definitions

We adopted the following definitions for HBV status, explanatory variables, and key study measures:

- HBV infection (outcome variable): Defined as the presence of HBsAg and/or anti-HBc with the absence of anti-HBs (Table 2).
- Serological markers (Table 2):
  ‒ Isolated anti-HBc positivity: Presence of anti-HBc in the absence of HBsAg and anti-HBs.
  ‒ Vaccine-induced immunity: Presence of anti-HBs in the absence of anti-HBc.
- Explanatory variables: Socio-demographic characteristics, lifestyle factors, comorbidities, HBV vaccination status, contact with infected family members, and in-patient healthcare exposure.
- Vaccine uptake: Proportion of study participants who self-reported having received HBV vaccination.
- Household definitions:
  ‒ Household: A residential unit with a unique address. Residential institutions, such as boarding schools, dormitories, hostels, or prisons,
  ‒ Household member: An individual living in the same dwelling or living unit.
- Prevalence of HBV infection: The proportion of individuals with HBV infection identified during the study period among the study population.
- Odds ratio (OR): Measure of association between exposures and HBV infection, defined as the ratio of the odds of exposure among infected individuals (HBsAg+) to the odds of exposure among non-infected individuals (HBsAg−). The odds were calculated as the probability of observing the exposure divided by the probability of not observing the exposure.
- Age groups: Participants were categorized into two age groups (<20 years and ≥20 years) to reflect exposure to the national hepatitis B vaccination policy. As infant HBV vaccination was introduced in Tunisia in 1995, individuals <20 years at the time of the survey were considered likely to have been eligible for routine infant vaccination, whereas older individuals were born before its implementation [9].
- Hospital follow-up: Self-reported regular medical care in a hospital setting for any diagnosed health condition. This includes individuals who reported being followed in hospital services for monitoring or treatment of a disease, whether through outpatient consultations or hospitalization.
- Endemicity classification (based on HBsAg prevalence) [5]:
  ‒ High endemicity: HBsAg prevalence > 8%.
  ‒ Intermediate endemicity: HBsAg prevalence 2–8%.
  ‒ Low endemicity: HBsAg prevalence < 2%.
- Vaccine effectiveness (VE): Assessed in two aspects:
  ‒ Serological vaccine efficacy (immunogenicity): Presence of protective anti-HBs antibodies following vaccination.
  ‒ Clinical vaccine effectiveness: Measured using self-reported vaccination status and HBV infection outcome:
    ○ Crude VE: VE = 1-OR, where OR is the odds ratio between HBV infection and self-reported vaccination.
    ○ Adjusted VE: VE = 1-aOR, where aOR is the odds ratio adjusted for relevant covariates.

### 2.5. Data Collection and Laboratory Testing

Data were collected using standardized, face-to-face questionnaires administered in Arabic by 24 trained healthcare worker teams. The questionnaires included detailed information on socio-demographic characteristics, potential risk factors for HBV transmission—including blood transfusions, sexual practices, hospitalizations, and drug use—and self-reported HBV vaccination history (Supplementary Materials).

Blood specimens were collected at primary healthcare centers from all participants and tested for hepatitis B virus markers, including hepatitis B surface antigen (HBsAg), hepatitis B surface antibody (anti-HBs), and hepatitis B core antibody (anti-HBc). Serological analyses were performed using the electrochemiluminescence immunoassay (ECLIA) method on the automated cobas e411 analyzer using the Elecsys HBsAg II, Elecsys Anti-HBs II, and Elecsys Anti-HBc II assay kits (Roche Diagnostics, Mannheim, Germany).

Although multiple HBV serological markers were measured, the present analysis focuses on HBsAg as a marker of active infection; analyses of other biomarkers are reported separately.

All samples were handled according to standardized protocols. Specimens were properly labeled, stored at appropriate temperatures, and transported to the laboratory within recommended time frames to preserve sample integrity. Quality control measures included the use of standard operating procedures for sample collection, processing, and testing, as well as periodic validation of assay performance to ensure the accuracy and reliability of results.

### 2.6. Data Management and Data Analysis

The analysis accounted for the complex survey design by incorporating sampling weights and design characteristics to obtain nationally representative estimates of HBV infection. Sampling weights were calculated to reflect the multistage stratified cluster sampling design and the unequal probability of selection across clusters. The weight assigned to each household was proportional to the number of households represented by the selected cluster in the population, taking into account differences in cluster size. The sample was further adjusted for the overall response rate to correct for potential non-response bias. Weighted prevalence estimates and corresponding 95% confidence intervals (CIs) were calculated for the overall population and stratified by region, age group, sex, and potential exposure factors.

#### 2.6.1. Descriptive Analysis

Quantitative variables were summarized using means and standard deviations or medians and interquartile ranges, depending on distribution. Categorical variables were summarized as frequencies and percentages, with binomial confidence intervals used to quantify uncertainty.

#### 2.6.2. Univariate Analysis

Associations between HBV infection and explanatory variables were assessed using Pearson’s Chi-square test or Fisher’s exact test, as appropriate. Crude odds ratios (ORs) with 95% CIs were estimated using logistic regression.

#### 2.6.3. Multivariate Analysis

Variables with a *p*-value ≤ 0.2 in univariate analysis, as well as potential confounders, were included in a backward stepwise binary logistic regression model to identify independent predictors of HBV infection. Model assumptions, including sample size adequacy and collinearity, were verified. Model fit was assessed using the Hosmer–Lemeshow goodness-of-fit test. Variables with a *p*-value ≤ 0.05 in the final model were considered statistically significant, and adjusted odds ratios (aORs) with 95% CIs were reported to indicate the strength of associations.

Data entry was performed using EpiData 3.1 software, and statistical analyses were conducted using SPSS version 20.

### 2.7. Ethical Considerations

The study protocol was approved by the Higher Council of Statistics, the National Ethics Committee of the Pasteur Institute of Tunis, and the National Instance for the Protection of Personal Data. Prior to participation, all individuals received an information note detailing the objectives and procedures of the survey. Written informed consent was obtained from all participants, or from parents/legal guardians in the case of minors, on the day of data collection.

Both the information notes and consent forms were provided in Arabic, the native language. Participation was entirely voluntary, and all individuals had the right to refuse without any consequences. Confidentiality and anonymity were strictly maintained throughout the study.

Participants with positive serological results were provided with feedback and appropriate support. Serological results were communicated to relevant healthcare structures via regional coordinators. Individuals identified with an HBV infection profile were referred to gastroenterology services for further clinical evaluation and medical follow-up. Immediate transaminase testing was performed, and serological monitoring, including HBsAg testing after six months, was planned for these individuals.

## 3. Results

### 3.1. Description of the Study Population

**Figure 2:**
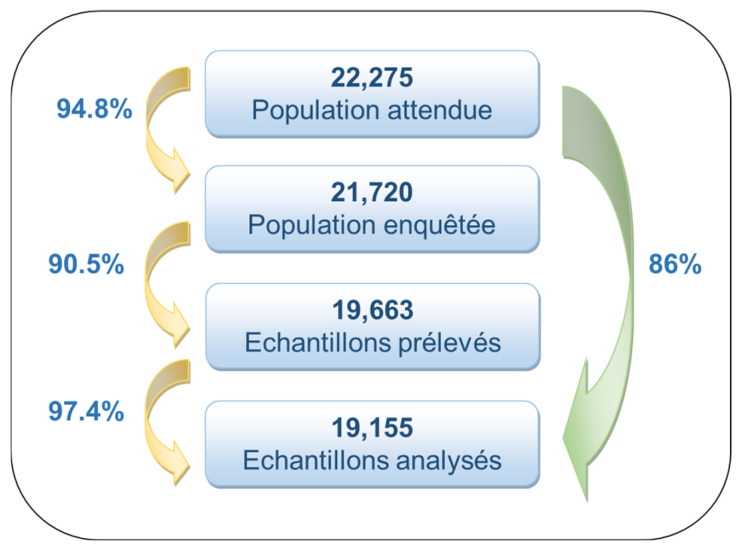
Data flow diagram selection of participants; seroprevalence survey of hepatitis B infection in Tunisia, 2014–2015.

According to the sampling plan developed by the National Institute of Statistics (INS), a total of 22,275 individuals were initially targeted for inclusion in the survey (Figure 2). The final sample was adjusted to account for the implemented sampling design and the observed response rate.

All selected households were visited, and household members meeting the inclusion criteria were invited to participate. Individuals who were absent during the initial visit were revisited within the same month to maximize participation. Non-respondents included individuals who refused to participate, those who did not provide blood samples, and those unavailable during both visits.

After accounting for non-response and sample adjustments, the final sample included 19,155 participants, representing an overall response rate of 86.0%. The sample remained representative of the Tunisian population in terms of age, sex, and geographic distribution, after applying sampling weights and adjustments for non-response.

This final dataset was used for all subsequent analyses, including estimation of HBV prevalence, assessment of risk factors, and evaluation of vaccine effectiveness (Figure 2).

### 3.2. Sociodemographic Characteristics

Among the 19,155 individuals tested, 11,830 (61.8%) were female, yielding a female-to-male sex ratio of 1.62. Most participants, 19,109 (78.4%), were aged over 20 years (Table 3).

Geographically, the highest proportion of participants, 9655 (50.4%), resided in the North region, followed by 6782 (35.4%) in the Central region. Approximately two-thirds of participants, 12,796 (66.8%), lived in urban areas, reflecting the national population distribution (Table 3).

Regarding marital status, 10,383 (73.9%) of participants were married. Educational level varied: 6515 (33.5%) had completed primary education and 4002 (11.1%) secondary education, while 3644 (19.6%) had never attended school.

With respect to professional activity, 10,007 participants (68.1%) reported having no socio-professional occupation (Table 3).

### 3.3. HBV Bio-Marker Prevalence

#### 3.3.1. HBsAg Prevalence

The overall prevalence of HBsAg in the study population was 1.7% (95% CI: 1.55–1.85%). HBsAg prevalence was significantly higher among males, at 2.1% (95% CI: 1.9–2.4%), compared to 1.4% (95% CI: 1.3–1.6%) among females, resulting in a male-to-female sex ratio of 1.48 (*p* < 0.001) (Table 4).

**Figure 3:**
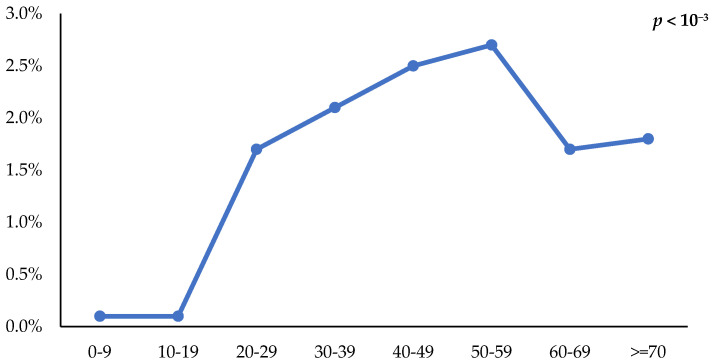
Prevalence of HBsAg by age group; seroprevalence survey of hepatitis B infection in Tunisia, 2014–2015 (N = 19,155).

The mean age of HBsAg-positive individuals was 48 ± 15.7 years. The prevalence of HBV infection increased with age, peaking at 2.7% in the 50–59-year age group, and then declined in older age groups (*p* < 0.001) (Figure 3).

The prevalence of HBsAg varied by region, with the highest rates observed in the Central and Southern regions, at 2.3% (95% CI: 2.0–2.7%) and 2.2% (95% CI: 1.8–2.8%), respectively.

**Figure 4:**
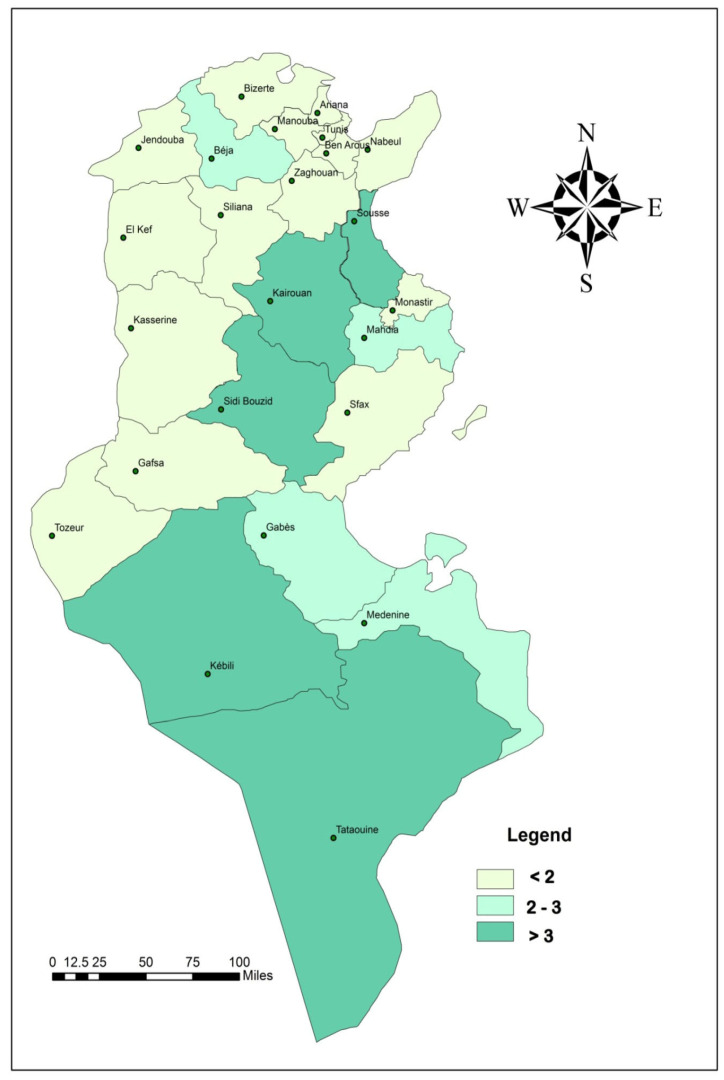
Geographic distribution of HBsAg prevalence in Tunisia; seroprevalence survey of hepatitis B infection in Tunisia, 2014–2015.

When stratified by area of residence, HBsAg prevalence was higher in rural areas (1.9%; 95% CI: 1.6–2.2%) compared to urban areas (Table 4, Figure 4).

#### 3.3.2. Factors Associated with Hepatitis B Infection: Univariate Analysis

In univariate analysis, HBV infection was significantly associated with several demographic, behavioral, and clinical factors. Individuals aged over 20 years had markedly higher odds of infection (OR = 36.63; 95% CI: 13.78–97.36; *p* < 0.001). Male sex was also associated with increased risk (OR = 1.48; 95% CI: 1.23–1.79; *p* < 0.001) (Table 5).

Traditional circumcision (OR = 3.20; 95% CI: 2.06–4.56; *p* < 0.001) and having an HBsAg-infected parent (OR = 2.63; 95% CI: 1.97–3.51; *p* < 0.001) were strongly associated with infection. Geographic factors were important: residing in the Central (OR = 2.11; 95% CI: 1.72–2.60; *p* < 0.001) or Southern region (OR = 2.03; 95% CI: 1.54–2.67; *p* < 0.001) increased the risk (Table 5).

Several behavioral and healthcare-related exposures were also significant predictors: shaving at a barbers (OR = 1.65; 95% CI: 1.25–2.17; *p* < 0.001), scarification practices (OR = 1.53; 95% CI: 1.21–1.93; *p* < 0.001), hospital follow-up (OR = 1.58; 95% CI: 1.30–1.92; *p* < 0.001), medical injections (OR = 1.46; 95% CI: 1.07–1.99; *p* < 0.001), dental procedures (OR = 1.29; 95% CI: 1.05–1.58; *p* = 0.012), history of invasive procedures during hospitalization (OR = 1.25; 95% CI: 1.02–1.52; *p* = 0.002), and surgical interventions (OR = 1.23; 95% CI: 1.01–1.50; *p* < 0.001). Professional activity was associated with a modestly increased risk (OR = 1.33; 95% CI: 1.09–1.61; *p* = 0.005) (Table 5).

Water source also appeared protective: individuals with a supply of tap water from the National Company for Water Distribution had lower odds of infection (OR = 0.60; 95% CI: 0.43–0.85; *p* = 0.004) (Table 5).

Vaccination was strongly protective. Participants reporting HBV vaccination had a significantly lower risk of infection (OR = 0.11; 95% CI: 0.07–0.18; *p* < 0.001), corresponding to a crude vaccine effectiveness (VE) of 89% (95% CI: 82–93%) (Table 5).

#### 3.3.3. Factors Associated with Hepatitis B Infection: Multivariate Analysis

In multivariate logistic regression analysis, several factors remained independently and significantly associated with HBV infection. Individuals aged over 20 years had substantially higher odds of infection (aOR = 15.10; 95% CI: 4.79–47.64; *p* < 0.001). Having a family member who is an HBV carrier was also a strong predictor (aOR = 2.82; 95% CI: 2.09–3.79; *p* < 0.001) (Table 6).

Geographic region was significantly associated with HBV infection, with residents of the Southern region (aOR = 2.51; 95% CI: 1.76–3.57; *p* < 0.001) and Central region (aOR = 2.18; 95% CI: 1.76–2.71; *p* < 0.001) having higher odds of infection compared to those in the Northern region. Male gender was independently associated with increased risk (aOR = 1.69; 95% CI: 1.39–2.05; *p* < 0.001), as was a history of hospital follow-up (aOR = 1.23; 95% CI: 1.01–1.51; *p* = 0.039) (Table 6).

HBV vaccination was the only protective factor identified in the multivariate model. Vaccinated individuals had significantly lower odds of infection (aOR = 0.35; 95% CI: 0.20–0.62; *p* < 0.001), corresponding to an adjusted vaccine effectiveness of 65% (adjusted VE = [1 − 0.35] × 100) compared to unvaccinated individuals (Table 6).

## 4. Discussion

This study aimed to assess the national prevalence of HBsAg in Tunisia. A total of 22,275 individuals were initially enrolled, of whom 19,155 provided blood samples, resulting in an 86% response rate. The national prevalence of HBsAg was 1.7% (95% CI: 1.55–1.85%), with higher prevalence observed among males, individuals over 20 years of age, and residents of the Central and Southern regions. Factors independently associated with HBV infection included male gender, age over 20 years, residency in the Southern or Central region, having a family member who is an HBV carrier, and hospital follow-up. Vaccination against HBV emerged as a strong protective factor, significantly reducing the risk of infection.

The observed national HBsAg prevalence of 1.7% represents a substantial decrease compared to previous studies, which classified Tunisia as an intermediate-endemic country with HBsAg prevalence of 5.3% (95% CI: 4.8–5.8%) [4,5]. This shift reclassifies Tunisia as a low-endemic country and highlights the long-term impact of universal HBV vaccination. Differences in diagnostic assays (ELISA in earlier studies versus ECLIA in the present study) may influence the comparability of prevalence estimates. However, electrochemiluminescence immunoassays are generally considered more sensitive in detecting low-level HBsAg, with discrepancies between ELISA and ECLIA largely attributed to differences in analytical sensitivity, particularly for weakly positive samples [13]. In this context, the observed decline in HBsAg prevalence is unlikely to be explained by reduced assay performance and more likely reflects a true epidemiological decrease, particularly in the setting of widespread vaccination. Similar trends have been observed in Morocco, where HBsAg prevalence dropped from intermediate- to low-endemic levels (1.81%) nearly a decade after the introduction of the vaccine [14,15,16].

The higher prevalence of HBV infection among males aligns with data from Egypt, where prevalence was 3.75% among males versus 2.2% among females (16). This gender disparity may reflect differences in exposure risk, biological susceptibility, healthcare access, and social behaviors. Risk factors such as injection drug use, men who have sex with men (MSM), and occupations with exposure to blood or waste are more common among men, contributing to the higher observed prevalence [17,18].

Age-specific prevalence was higher among individuals older than 20 years, likely reflecting the historical implementation of the HBV vaccination program, which was introduced approximately 20 years ago and therefore did not directly protect this age group. Indirect benefits through herd immunity may contribute to some protection in this population. Similar findings were reported in Taiwan, where universal HBV vaccination significantly reduced HBsAg and anti-HBc prevalence among university students two decades post-implementation [19].

Geographically, our study revealed a clear North–South gradient, with the highest HBsAg prevalence observed in the Southern and Central regions. Governorates including Sidi Bouzid, Kairouan, Tataouine, and Kebili had prevalences exceeding 3%, while Mahdia and Beja were classified as intermediate-endemic. These findings demonstrate a marked decrease in HBsAg prevalence compared to pre-vaccination data; for example, that in Tataouine decreased from 5.6% in 1996 to 3% in 2015, and that in Beja from 4.2% to 2%. No hyperendemic governorates were identified, further supporting the effectiveness of the national vaccination program [4].

Having a family member who is an HBV carrier was associated with an increased risk of infection (aOR = 2.8; 95% CI: 2.09–3.79; *p* < 0.001), consistently with previous studies highlighting household transmission as a key factor [20,21,22]. Similarly, hospital follow-up was significantly associated with HBV infection (aOR = 1.23; 95% CI: 1.01–1.51; *p* = 0.039), reflecting potential exposure to nosocomial transmission when hygiene and sterilization protocols are not strictly adhered to [23].

HBV vaccination was strongly protective. Vaccinated participants had a 65% lower likelihood of infection compared to unvaccinated individuals (aOR = 0.35; 95% CI: 0.20–0.62; *p* < 0.001). This aligns with global evidence demonstrating that higher vaccination coverage corresponds with lower HBV prevalence, as observed in Tanzania, Nigeria, and China [24,25,26]. In the same study population, vaccine-induced immunity was observed in 64.0% of vaccinated individuals, as reported elsewhere, further supporting the long-term effectiveness of the vaccination program [27].

### Strengths and Limitations

This study provides the first large-scale, nationally representative household-based estimate of HBV infection prevalence and associated factors in Tunisia. Previous studies were limited to selected populations (e.g., blood donors, hemodialysis patients, newborns) or restricted geographic areas [10,28,29,30]. Our study included 19,155 tested participants across all ages, sexes, and regions, conducted 20 years after the introduction of universal HBV vaccination, providing a unique long-term perspective on the program’s impact.

Importantly, these findings establish a robust national baseline for monitoring progress toward hepatitis B elimination in Tunisia, in alignment with WHO recommendations and global targets for viral hepatitis elimination.

High-quality serological testing was performed using electrochemiluminescence immunoassay (ECLIA) on the Cobas e411 platform, ensuring high sensitivity and specificity. Multivariate analysis allowed adjustment for confounders, enhancing the reliability of our findings.

However, several limitations must be acknowledged. As this is a cross-sectional study, causal relationships cannot be definitively established. Recall bias may have affected self-reported exposures, including vaccination history and at-risk behaviors, potentially underestimating associations with HBV infection. Social desirability bias could have led to under-reporting of sensitive behaviors such as injection drug use or sexual practices. Certain risk factors reported in the literature, including tattooing and acupuncture, were not significantly associated with HBV infection in our study [31,32].

Blood transfusion, historically a significant risk factor, was not associated with infection in this study, likely reflecting improved blood screening practices [33]. Co-infections with other bloodborne pathogens, such as HIV or HCV, were not assessed, which may influence immune response and disease progression. Finally, self-reported vaccination status may be affected by recall bias, potentially underestimating the protective effect of vaccination.

Despite these limitations, this study provides robust, nationally representative data on HBV prevalence, risk factors, and vaccine effectiveness, offering critical evidence to guide public health policy and HBV prevention strategies in Tunisia.

## 5. Conclusions and Recommendations

This study provides the first nationally representative estimate of HBV seroprevalence in the general population of Tunisia in 2014–2015. The national HBsAg seroprevalence was 1.7%, reflecting a substantial decline compared to previous estimates and reclassifying Tunisia from an intermediate- to a low-endemic country. These findings highlight the significant impact of the HBV vaccination program implemented in 1995 in reducing the burden of infection.

Our results underscore the importance of sustaining and enhancing vaccination programs, with targeted efforts toward high-risk groups, including males, adults over 20 years, residents of the Central and Southern regions, household contacts of HBV-infected individuals, and individuals with frequent hospital follow-up.

In addition, promoting strict infection prevention and control measures in healthcare settings, implementing intrafamilial precautions to prevent blood-borne transmission, and conducting targeted screening of high-risk populations are essential strategies for the long-term control of HBV infection in Tunisia.

## Figures and Tables

**Table 1:** Sample size calculation by region; seroprevalence survey of hepatitis B infection in Tunisia, 2014–2015.

Region	Prevalence (p)	Precision (d)	Minimal Sample Size	Expected Sample Size
North	2.4%	0.4%	8784	10,540
Center	0.8%	0.3%	5291	6348
South	0.6%	0.3%	3976	4771
**Total**			**18,051**	**21,660**

**Table 2:** Interpretation of test results for hepatitis B virus infection; seroprevalence survey of hepatitis B infection in Tunisia, 2014–2015 [12].

HBsAg	Total Anti-HBc	Anti-HBs	Interpretation
+	+/−	–	HBV infection
–	–	+	Vaccine induced immunity
–	+	–	Isolated anti-HBc positive

**Table 3:** Description of study population by socio-demographic characteristics; seroprevalence survey of hepatitis B infection in Tunisia, 2014–2015 (Nsample = 19,155).

	Number	Percent
Gender (n = 19,155)		
Male	7325	38.2%
Female	11,830	61.8%
Age by 10-year range (n = 19,155)		
[0–9]	1570	8.2%
[10–19]	2560	13.4%
[20–29]	2452	12.8%
[30–39]	2973	15.5%
[40–49]	2909	15.2%
[50–59]	2792	14.6%
[60–69]	2114	11.0%
≥70	1739	9.1%
NP	46	0.2%
Age group (years) (n = 19,155)		
≤20	4133	21.6%
>20	14,976	78.2%
NP	46	0.2%
Region of residence (n = 19,155)		
North	9655	50.4%
Center	6782	35.4%
South	2718	14.2%
Area of residence (n = 19,155)		
Urban	12,796	66.8%
Rural	6359	33.2%
Marital status (n = 19,155)		
Single	3325	17.4%
Married	10,383	52.4%
Divorced/separated/widowed	338	1.8%
NP	5109	26.7%
Level of education (n = 19,155)		
Never Enrolled	3644	19.0%
Preschool	547	2.9%
Primary	6515	34.0%
Middle school	2054	10.7%
High school	4002	20.9%
University	1799	9.4%
NP	594	3.1%
Professional activity (n = 19,155)		
Yes	4680	31.9%
No	10,007	68.1%
NP	4468	23.3%

**Table 4:** Distribution of HBsAg prevalence by selected demographic characteristics; seroprevalence survey of hepatitis B infection in Tunisia, 2014–2015 (Nsample = 19,155).

	Positive HBsAg (n = 348)	Prevalence (%)	95% CI Lower Upper	
Gender (n = 19,155)				
Male (n = 7325)	168	2.1%	1.9%	2.4%
Female (n = 11,830)	180	1.4%	1.3%	1.6%
Age group (years) (n = 19,109)				
≤20 (n = 4133)	3	0.1%	0.0%	0.2%
>20 (n = 14,976)	345	2.1%	2.0%	2.3%
Region of residence (n = 19,155)				
North (n = 9655)	121	1.1%	1.0%	1.3%
Center (n = 6782)	167	2.3%	2.0%	2.7%
South (n = 2718)	60	2.2%	1.8%	2.8%

**Table 5:** Individual characteristics associated with HBV infection. Univariate analysis (seroprevalence survey of hepatitis B infection in Tunisia, 2014–2015 (Nsample =19,155)).

	N Sample	OR	95% CI Lower Upper		*p*-Value
Gender (n = 19,155)					
Female	11,830	Ref			
Male	7325	1.483	1.23	1.78	<10^−3^
Age group (years)					
≤20	4133	Ref			
>20	14,976	36.62	13.78	97.36	<10^−3^
Region of residence (n = 19,155)					
North	9655	Ref			
Center	6782	2.11	1.72	2.60	<10^−3^
South	2718	2.03	1.54	2.67	<10^−3^
Area of residence (n = 19,155)					
Urban	12,796	Ref			
Rural	6359	1.16	0.954	1.41	0.135
Marital status (n = 14,046)					
Single	3325	Ref			
Married	10,383	1.252	0.976	1.606	0.076
Divorced/separated/widowed	338	1.348	0.718	2.531	0.351
Level of education (n = 18,561)					
Middle school level	2054	Ref			
Preschool	547	1.660	0.904	3.049	0.102
Primary level	6515	1.531	1.068	2.195	0.021
Never enrolled	3644	1.338	0.906	1.975	0.143
High school level	4002	1.205	0.812	1.787	0.353
University level	1799	1.651	1.068	2.550	0.024
Professional activity (n = 14,687)					
No	10,007	Ref			
Yes	4680	1.325	1.089	1.611	0.005
Water supply (n = 18,453)					
Other source	16,471	Ref			
Tap water	1982	0.603	0.428	0.85	0.004
Waste water disposal (n = 18,418)					
Water sewage system	11,548	Ref			
Other	6870	1.068	0.879	1.297	0.505
Family member with HBV (n = 17,906)					
No	16,138	Ref			
Yes	977	2.633	1.972	3.514	<10^−3^
I don’t know	791	1.604	1.086	2.369	0.018
Sharing personal items (n = 15,207)					
No sharing	3342	Ref			
Sharing of at least one personal object (toothbrush, razor blade, scissors or clippers)	11,865	1.178	0.929	1.495	0.174
Traveling abroad (n = 15,126)					
No	13,531	Ref			
Yes	1595	0.872	0.651	1.169	0.357
Shaving at a barbers (n = 7042)					
No	2902	Ref			
Yes	4140	1.653	1.256	2.175	<10^−3^
Sexual orientation (n = 14,375)					
Prefer not to respond	3355	Ref			
Not applicable	1913	1.022	0.736	1.420	0.896
Heterosexual	8996	1.000	0.797	1.255	0.999
Homosexual	111	1.515	0.540	4.249	0.429
Sexual partner (n = 14,841)					
None	2519	Ref			
Single partner	9215	1.137	0.856	1.511	0.373
Multiple partners	906	1.611	1.062	2.445	0.025
Prefer not to respond	2201	1.603	0.745	1.516	0.736
Scarification (n = 18,654)					
No	15,963	Ref			
Yes	2691	1.533	1.217	1.930	<10^−3^
Tattooing (n = 18,686)					
No	17,844	Ref			
Yes	842	1.065	0.688	1.651	0.775
IDU (Injecting drug use) (n = 15,218)					
No	15,115	Ref			
Yes	103	1.572	0.660	3.748	0.305
Piercing (n = 18,616)					
No	13,362	Ref			
Yes	5254	0.355	0.063	1.784	0.198
Acupuncture (n = 18,400)					
Yes	151	Ref			
No	18,249	0.355	0.063	1.784	0.198
Circumcision (n = 6912)					
Medical	2300	Ref			
Traditional	4612	3.067	2.061	4.564	<10^−3^
Location of circumcision (n = 6907)					
Hospital/clinic	967	Ref			
Other	5940	12.926	3.886	43.000	<10^−3^
Dental procedures (n = 19,043)					
No	7576	Ref			
Yes	11,467	1.293	1.058	1.581	0.012
Surgical intervention (n = 18,705)					
No	13,016	Ref			
Yes	5689	1.236	1.016	1.505	0.033
Blood transfusion (n = 15,181)					
After 1992	673	Ref			
Before 1992	245	0.726	0.441	1.193	0.205
Never	14,263	1.329	0.719	2.458	0.363
Blood donation (n = 15,393)					
No	11,973	Ref			
Yes	3420	1.109	0.892	1.380	0.351
Hospital follow-up (n = 19,064)					
No	14,155	Ref			
Yes	4909	1.587	1.306	1.928	<10^−3^
Medical injection (n = 18,867)					
No	2746	Ref			
Yes	16,121	1.465	1.073	1.998	0.016
Invasive procedure (n = 19,155)					
No	14,099	Ref			
Yes	5056	1.254	1026	1.533	0.027
Lower digestive endoscopy (n = 2750)					
No	2661	Ref			
Yes	89	2.707	1.027	7.133	0.043
HBV vaccine uptake (n = 18,893)					
Unvaccinated *	13,967	Ref			
Vaccinated	4926	0.11	0.07	0.18	<10^−3^

Abbreviations: CI: confidence interval; OR: crude odds ratio; Ref: reference. Unvaccinated *: category includes also don’t know for the univariate analysis.

**Table 6:** Multivariate analysis of the factors associated with HBV infection: final model (seroprevalence survey of hepatitis B infection in Tunisia, 2014–2015 (Nsample = 19,155)).

	aOR	95% CI		*p*-Value
		Lower	Upper	
Gender				
Female	Ref			
Male	1.695	1.398	2.056	<10^−3^
Age group (years)				
≤20	Ref			
>20	15.107	4.790	47.644	<10^−3^
Region of residence				
North	Ref			
Center	2.186	1.762	2.713	<10^−3^
South	2.513	1.892	3.337	<10^−3^
Family member with HBV infection				
No	Ref			
Yes	2.823	2.098	3.799	<10^−3^
I don’t know	1.504	1.006	2.247	0.047
Hospital follow-up (n = 19,064)				
No	Ref			
Yes	1.236	1.011	1.512	0.039
Vaccine uptake (n = 18,893)				
Unvaccinated *	Ref			
Vaccinated	0.355	0.202	0.624	<10^−3^

Abbreviations: CI: confidence interval; aOR: adjusted odds ratio; Ref: reference category. *: Unvaccinated category also includes don’t know for the multivariate analysis. Hosmer–Lemeshow test *p*-value ≤ 0.05.

## Data Availability

Data is unavailable due to privacy or ethical restrictions.

## Supplementary Materials

The following supporting information can be downloaded at https://www.mdpi.com/article/10.3390/vaccines14050373/s1: National_Survey_Prevalence of VHB_Tunisia_Individuals_Adults_Survey. National_Survey_Prevalence of VHB_Tunisia_Individuals__Minors_Survey. National_Survey_Prevalence of VHB_Tunisia_Household_Survey.

## Abbreviations

The following abbreviations are used in this manuscript:

Anti-HBC	Antibody against the hepatitis B core antigen
Anti-HBs	Antibody against the hepatitis B surface antigen
Anti-HBe	Antibody against the hepatitis B e antigen
CI	Confidence interval
HCC	Hepatocellular carcinoma
HBsAg	Hepatitis B surface antigen
HBV	Hepatitis B virus
HCV	Hepatitis C virus
HIV	Human Immunodeficiency Virus
ECLIA	Electrochemiluminescence immunoassay
NIS	National Institute of Statistics
ONMNE	National Observatory of New and Emerging Diseases
OR	Odds ratio
WHO	World Health Organization
