# Supplementary material for: National Prevalence and Risk Factors of Hepatitis B Virus Infection in Tunisia Two Decades After Vaccine Introduction
Source: Vaccines (Basel). 2026 Apr 23;14(5):373. doi: 10.3390/vaccines14050373 (PMC13211386; doi:10.3390/vaccines14050373)
Supplement: Supplementary file 1 [file vaccines-14-00373-s001.zip › 20141215_National_Survey_Prevalence of VHB_Tunisia_Household_Survey_English Version.pdf]

Entry Number : └┴┴┘

HOUSEHOLD FORM

Household Unique Identifier: └┴┘ └┴┘ └┴┘

Date of Household Inclusion in the Survey └┴┴┴┴┴┴┴┘ D D M M Y Y Y Y

HOUSEHOLD IDENTIFICATION

Gouvernorate └┴┘ Delegation └┴┘ Imadat └┴┘

Geo referrring ◯ (1 Yes, 2 No) If yes, precise Date └┴┴┴┴┴┴┴┘ D D M M Y Y Y Y

GPS reference └┴┘ Exported GPS File Name---

DEMOGRAPHIC DATA		
**Q1**	Number of families in the household	└┘
**Q2**	Number of individuals in the household	└┴┘
HOUSING		
**Q3**	Where do you live ?<br>Arab house<br>Villa or villa floor<br>Apartment<br>Rudimentary<br>Others<br>ND	 ◯1<br>◯2<br>◯3<br>◯4<br>◯5 Specify ---<br>◯9
**Q4**	Number of rooms (excluding kitchen and bathroom)	└┴┘

Household Unique Identifier: └┴┘ └┴┴┘ └┴┴┘

WATER SUPPLY AND WASTEWATER DISPOSAL		
Q5	How do you obrain your water supply ?<br>Running water<br>Well, Spring<br>Citern, public fountain<br>Other<br>ND	◯1<br>◯2<br>◯3<br>◯4 Specify --<br>◯9
Q6	What is your wastewater disposal system ?<br>Sewer network<br>Septic tank<br>Latrine<br>Soak pit<br>Other<br>ND	◯1<br>◯2<br>◯3<br>◯4<br>◯5 Specify --<br>◯9

ENVIRONMENT		
Q7	Presence of animal shelters	◯ (1 Yes, 2 No)
Q8	Presence of wells for irrigation	◯ (1 Yes, 2 No)
Q9	Walls without finishing/plastering	◯ (1 Yes, 2 No)
