# Supplementary material for: National Prevalence and Risk Factors of Hepatitis B Virus Infection in Tunisia Two Decades After Vaccine Introduction
Source: Vaccines (Basel). 2026 Apr 23;14(5):373. doi: 10.3390/vaccines14050373 (PMC13211386; doi:10.3390/vaccines14050373)
Supplement: Supplementary file 1 [file vaccines-14-00373-s001.zip › 20141215_National_Survey_Prevalence of VHB_Tunisia_Individual_Adults_Survey_English_Version.pdf]

Entry Number └┴┴┴┴┘

ANONYMOUS DATA COLLECTION FORM
INDIVIDUAL RECORDS

ADULTS OVER THE AGE OF 18 ONLY

Individual Identification

Unique Identifier: (UI) :

└┴┘ └┴┘ └┴┘ └┘ └┴┘

Deelegation code Family code N° of individual

Name and surname of the individual :

PART ONE: SOCIO-DEMOGRAPHIC DATA

1. SUBJECT IDENTIFICATION

N°	Questions	Answers	Skip
Q1	Gender	◯1 male 2 Female	
Q2	Date of birth<br>(If unknown, specify age)	D D M M Y Y Y Y<br>years	
Q3	Date of survey	D D M M Y Y Y Y	
Q4	Gouvernorate of residence		
Q4	Delegation of residence		
Q5	Exact Address<br>N° Phone number	--<br>--<br>--	
Q6	Have you lived here all your life?	◯(1 Yes, 0 No)<br>if No	Q8
Q7	If No, how long have you lived here?<br>(Don't know / no response code 999)	years	

2. SOCIAL COVERAGE

Q8	Are you a beneficiary of CNAM?:	◯ (1 Yes, 0 No)<br>if No	Q9
	If yes specify	◯1 Public healthcare system<br>◯2 Private healthcare system<br>◯3 Reimbursement system	
Q9	Are you a beneficiary of Free Medical Assistance (AMG)?	◯ (1 Yes, 0 No)	
	If Yes, specify type	◯1 AMG type1<br>◯2 AMG type 2	
Q10	Are you enrolled in supplementary health insurance?	◯ (1 Yes, 0 No)	

3. MARITAL STATUS, EDUCATION, AND OCCUPATION			
**N°**	**Questions**	**Answers**	**Skip**
**Q11**	What is your marital status?	◯1 Single<br>◯2 Married<br>◯3 Divorced<br>◯4 Separated<br>◯5 Widowed<br>◯9 Not applicable	
**Q12**	What is your level of education? (Highest level reached, or current if still studying)	◯0 Never attended school<br>◯1 Kotteb, preschool<br>◯2 Primary<br>◯3 Middle school<br>◯4 High school<br>◯5 University<br>◯9 Not applicable	
**Q13**	Do you currently have a professional activity?	◯ (1 Yes, 0 No) If Yes<br>If no	Q14-15<br>Q16
**Q14**	If Yes, what is your current profession?	--<br>--	
**Q15**	If Yes, what is your sector of activity?	◯1 Agriculture & Fishing<br>◯2 Manufacturing<br>◯3 Mining & Energy<br>◯4 Construction & Public Works<br>◯5 Commerce<br>◯6 Transport, information and communication<br>◯ 7 Education, Health, Administrative Services & Defense<br>◯8 Household Services<br>◯9 Hospitality & Catering<br>◯10 Arts, Entertainment & Recreation<br>◯ 99 Other (Specify)---	
**Q16**	If you do not work, you are:	◯0 Never worked (Specify reason)----------------<br>---<br>◯1 Unemployed seeking first job<br>◯2 Still studying<br>◯3 Retired (previous profession)-----------------------------<br>---	
**Q17**	Have you completed military service? (Men only)	◯ (1 Yes, 0 No)	

4. STAYS OUTSIDE TUNISIA			
**N°**	**Questions**	**Answers**	**Skip**
**Q18**	Have you ever stayed abroad for more than 3 months?	◯ (1 Yes, 0 No) if No	Q 21
**Q19**	If Yes, where?	◯ 1 Northern Europe<br>◯ 2 Western Europe<br>◯ 3 Eastern Europe<br>◯ 4 Southern Europe<br>◯ 5 Middle East<br>◯ 6 North Africa<br>◯ 7 Sub-Saharan Africa<br>◯ 8 Asia<br>◯ 9 Indian Subcontinent<br>◯ 10 Pacific<br>◯ 11 North America<br>◯ 12 South America	
**Q20**	Did you receive medical care there?	◯ (1 Yes, 0 No, 9 I don't know)	
**5. FAMILY HABITS**			
**Q21**	Have you shared any of these items with another family member?<br>Toothbrush<br>Towel<br>Razor<br>Scissors / Nail clipper	(0 No, 1 Occasionally, 2 Frequently, 3 Always, 9 Don't know)<br>|__| |__| |__| |__|	
**Q22**	Do you shave at a barber? (Men only)	◯ (1 Yes, 0 No) If No	Q25
**Q23**	If Yes, how many times per month?	|__|__|	
**Q24**	Does the barber use a new razor each time?	◯ (1 Yes, 0 No, 9 I don't know)	
**6. BLOOD TRANSFUSION OR BLOOD DONATION**			
**Q25**	Have you ever had a blood transfusion?	◯ (1 Yes, 0 No, 9 Don't know) *If No*	Q29
**Q26**	Date(s) of transfusion (year)	|__|__|__|__| |__|__|__|__| |__|__|__|__|	
**Q27**	Where were you transfused?	--	
**Q28**	How many times?	|__|__|	
**Q29**	Have you ever donated blood?	◯ (1 Yes, 0 No, 9 Don't know) *If No*	Q33
**Q30**	Where did you donate blood?	--	
**Q31**	How many times?	|__|__|	
**Q32**	When did you donate blood?	|__|__|__|__| |__|__|__|__| |__|__|__|__|	

7. MEDICAL AND SURGICAL HISTORY			
**N°**	**Questions**	**Answers**	**Skip**
**Q33**	Are you being treated for any disease? If Yes, specify up to 3 chronic illnesses	◯ (1 Yes, 0 No, 9 Don't know)<br>Specify 1 -------------------------|__|__| Y|__|__| M<br>Specify 2-------------------------|__|__| Y |__|__| M<br>Specify 3 -------------------------|__|__| Y |__|__| M	
**Q34**	Are you hemophilic? If Yes, since when?	◯ (1 Yes, 0 No, 9 Don't know)<br>|__|__| year |__|__| month	
**Q35**	Are you on chronic dialysis?	◯ (1 Yes, 0 No, 9 Don't know)<br>|__|__| year |__|__| month	
**Q36**	Have you had an organ transplant?	◯ (1 Yes, 0 No, 9 Don't know)<br>|__|__| year |__|__| month	
**Q37**	Have you had surgery? If Yes, specify type and since when	◯ *(0 No, 1 Once, 2 More than once)* ***If No***<br>Type 1 -------------------------|__|__| Y|__|__| M<br>Type 2 -------------------------|__|__| Y|__|__| M<br>Type 3 -------------------------|__|__| Y|__|__| M	**Q39**
**Q38**	Where were your surgery/s performed?<br>Hospital<br>Private clinic<br>Other	*(1 Once, 2 More than once)*<br>|__| Specify --------------------------------<br>|__| Specify --------------------------------<br>|__| Specify --------------------------------	
**Q39**	Have you ever had an injection before?	◯ (1 Yes, 0 No, 9 Don't know) If ***No***	**Q44**
**Q40**	If Yes, how many injections in your life?	|_| Less than 10<br>|_| More than 10<br>|_| Regularly<br>|_| Don't know	
**Q41**	Usually, who administers your injections?<br><br>Doctor / Nurse / Pharmacist /<br>Other<br>health personnel /<br>Barber /<br>Other (Specify)	*(0 No, 1 Occasionally, 2 Frequently, 3 Always, 9 Don't know)*<br>|__| |__| |__| |__| |__| --	
**Q42**	Injections usually given with:<br>Open plastic syringe in front of you<br>Closed plastic syringe<br>Glass syringe	*(1 Yes, 0 No, 9 Don't know)*<br>|__| |__| |__|	
**Q43**	Type of injection:<br>injection SC<br>injection IM<br>injection IV	*(1 Yes, 0 No, 9 Don't know)*<br>|__| |__| |__|	
**Q44**	Have you been hospitalized? If Yes, specify hospital/clinic	◯ (1 Yes , 0 No) ***if no***<br>*specify* --	**Q49**
**Q45**	How many times?	|__|__|	
**Q46**	Year(s) of hospitalization	|__|__|__|__| |__|__|__|__| |__|__|__|__|	

N°	Questions	Answers	Skip
	**8. SURGICAL AND HOSPITAL PROCEDURES**		
Q47	Reason for hospitalization<br>Diagnosis & Treatment<br>Surgery<br>Pregnancy & Delivery<br>Other (Specify)	*(0 No, 1 Once, 2 More than once)*<br>|__| |__| |__| |__|---------------------------------------	
Q48	During hospitalization, did you have any of the following procedures?<br>Biopsy<br>Urinary catheterization<br>Blood catheterization<br>Hemodialysis<br>Coronary angiography<br>Upper digestive endoscopy<br>Lower digestive endoscopy<br>Bronchoscopy<br>Laparoscopy<br>Arthroscopy<br>Hysteroscopy (Women)<br>Sutures<br>Invasive radiology exams<br>Mesotherapy<br>Acupuncture<br>Varicose vein sclerotherapy<br>Other (Specify)	*(Nb of times, 0 Never)*<br><br>|__| |__| |__| |__| |__| |__| |__| |__| |__| |__| |__| |__| |__| |__| |__| |__| |__|--	
	**9. DENTAL TREATMENT**		
Q49	Have you received dental care?	◯ (1 Yes , 0 No) if no	Q52
Q50	If yes, specify :<br>Gum treatment<br>Scaling<br>Tooth extraction<br>Local anesthesia for extraction<br>Other (Specify)	*(0 No, 1 Once, 2 More than once)*<br>|__| |__| |__| |__| |__|--	
Q51	Usual place for dental care<br>Hospital dentist<br>Private dentist<br>Barber<br>Other (Specify))	*(0 No, 1 Once, 2 More than once)*<br>|__| |__| |__| |__|--	
	**10. DRUG USE**		
Q52	Have you ever used drugs nasally?	|__| *(0 Never, 1 Once, 2 More than once, 9 Prefer not to answer)*	
Q53	Have you ever used drugs intravenously??	|__| *(0 Never, 1 Once, 2 More than once, 9 Prefer not to answer)*	
Q54	Has any of your regular or occasional partners ever used IV drugs?	|__| *(0 Never, 1 Once, 2 More than once, 9 Prefer not to answer)*	
	**11. OCCUPATIONAL OR ACCIDENTAL RISK**		
Q55	Have you ever been pricked with a needle or material contaminated with blood or bodily fluids?	|__| *(0 Never, 1 Once, 2 More than once, 9 Don't know)*	
Q56	Have you ever had blood or bodily fluids contact your mucous membranes or broken skin?	|__| *(0 Never, 1 Once, 2 More than once, 9 Don't know)*	
	**12. TATTOO, SCARRING, EAR PIERCING, ACUPUNCTURE**		
Q57	Have you ever had a tattoo?	|__||__| *(Number of times (0 if never)*	
Q58	Have you ever had a scarification?	|__||__| *(Number of times (0 if never)*	
Q59	Have you ever had acupuncture ?	|__||__| *(Number of times (0 if never)*	
Q60	Have you ever had a piercing?	|__||__| (1 Yes , 0 No)	

13. HEPATITIS B HISTORY			
Q61	Have you been vaccinated against hepatitis B?	|__| (1 Yes, 0 No, 9 Don't know) if No	Q63
Q62	If Yes, how many doses?	|__|	
Q63	Have you ever had acute hepatitis B diagnosed by a physician?	|__| (1 Yes, 0 No, 9 Don't know) if No	Q65
Q64	If Yes, was HBs antigen still positive after infection?	|__| (1 Yes, 0 No, 9 Don't know)	
Q65	Do you know if a close relative is a chronic carrier of hepatitis B (HBs antigen)?	|__| (1 Yes, 0 No, 9 Don't know) if No	Q67
Q66	If Yes, what is their relationship to you	○ 1 Father<br>○ 2 Mother<br>○ 3 Brother<br>○ 4 Sister<br>○ 5 Partner/Spouse<br>○ 6 Child<br>○ 9 Other (Specify)	
Q67	Have you ever had a blood test for HBs antigen? If yes, specify context	|__| (1 Yes, 0 No, 9 Don't know) if No	Q86
Q68	During routine health check	|__| (1 Yes, 0 No, 9 Don't know)	
Q69	Before hepatitis B vaccination	|__| (1 Yes, 0 No, 9 Don't know)	
Q70	During blood donation	|__| (1 Yes, 0 No, 9 Don't know)	
Q71	During combined HIV-HBV screening	|__| (1 Yes, 0 No, 9 Don't know)	
Q72	Due to a risk factor	|__| (1 Yes, 0 No, 9 Don't know)	
Q73	Due to blood test showing liver anomaly (transaminases)	|__| (1 Yes, 0 No, 9 Don't know)	
Q74	During treatment of liver disease	|__| (1 Yes, 0 No, 9 Don't know)	
Q75	Due to partner's confirmed HBs positive	|__| (1 Yes, 0 No, 9 Don't know)	
Q76	Due to family member's confirmed HBs positive	|__| (1 Yes, 0 No, 9 Don't know)	
Q77	Other (Specify)---		
Q78	Have you ever had a negative result for this/these test(s)?	|__| (1 Yes, 0 No, 9 Don't know) if No	Q80
Q79	If Yes, date of last negative result	|__||__|month |__||__| Year	
Q80	Have you ever had a positive result for this/these test(s)?	|__| (1 Yes, 0 No, 9 Don't know) if No	Q82
Q81	If Yes, date of last positive result	|__||__|month |__||__| Year	
Q82	Are/were you followed by a physician for this infection?	|__| (1 Yes, 0 No) if No	Q85
Q83	If Yes, type of physician	1 General Practitioner, 2 Specialist, 3 Both	
Q84	If followed by a physician, location?	○ 1 In town, 2 Hospital, 3 Both	
Q85	Have you ever been told you had hepatitis B in the past and were cured?	|__| (1 Yes, 0 No, 9 Don't know)	

14. HEPATITIS C HISTORY			
**Q86**	Have you ever had hepatitis C serology (anti-HCV)? Specify context	|__| (1 Yes, 0 No, 9 Don't know) If No	Q103
**Q87**	During routine health check	|__| (1 Yes, 0 No, 9 Don't know)	
**Q88**	During blood donation	|__| (1 Yes, 0 No, 9 Don't know)	
**Q89**	During combined HIV-HCV screening	|_| ((Yes, 0 No, 9 Don't know)	
**Q90**	Due to a risk factor	|_| (1 Yes, 0 No, 9 Don't know)	
**Q91**	During blood test showing liver anomaly	|_| (1 Yes, 0 No, 9 Don't know)	
**Q92**	During liver disease treatment	|_| (1 Yes, 0 No, 9 Don't know)	
**Q93**	Due to partner's known positive HCV serology	|_| (1 Yes, 0 No, 9 Don't know)	
**Q94**	Due to family member's known positive HCV serology	|_| (1 Yes, 0 No, 9 Don't know)	
**Q95**	Other (Specify) ---		
**Q96**	Have you ever had a negative result for this/these test(s)?	|_| (1 Yes, 0 No, 9 Don't know) If No	Q98
**Q97**	If Yes, date of last negative result	|__||__|month |__||__| Year	
**Q98**	Have you ever had a positive result for this/these test(s)?	|__| (1 Yes, 0 No, 9 Don't know) If No	Q100
**Q99**	If Yes, date of last positive result	|__||__|month |__||__| Year	
**Q100**	Are/were you followed by a physician for this infection	|_| (1 Yes, 0 No) if No	Q103
**Q101**	If Yes, type of physician	1 General Practitioner, 2 Specialist, 3 Both	
**Q102**	If followed by a physician, location	◯1 In town, 2 Hospital,	
**Q103**	Do you know if a close relative had positive HCV serology?	|__| (1 Yes, 0 No, 9 Don't know) If No	Q105
**Q104**	If Yes, relationship to you	◯ 1 Father<br>◯ 2 Mother<br>◯ 3 Brother<br>◯ 4 Sister<br>◯ 5 Partner<br>◯ Spouse<br>◯ Child<br>◯ 9 Other (Specify)	

15. SEXUALITY			
Q105	Considering your entire sexual life:		
Q104	How do you identify yourself?	◯1 Never had sexual intercourse<br>◯2 Heterosexual<br>◯3 Homosexual<br>◯9 Prefer not to answer	
Q105	How many sexual partners have you had in your life?	◯0 None<br>◯1 One<br>◯3 Two to Nine<br>◯4 Ten or more<br>◯9 Prefer not to answer | |	
Q106	In the past 12 months, if you had sex with an occasional partner, did you use a condom or ask your partner to use one?	◯1 Always<br>◯2 Occasionally<br>◯3 Never<br>◯4 No occasional partner<br>◯9 Prefer not to answer	

16. CIRCUMCISION (MEN ONLY)			
Q107	At what age were you circumcised?	|__|__|YEARS	
Q108	Who performed your circumcision?	◯1 Doctor<br>◯2 Nurse<br>◯3 Traditional circumciser,<br>◯ 9 Don't know | |	
Q109	Where was your circumcision performed?	◯1 At home<br>◯2 Hospital<br>◯3 Private clinic,<br>◯4 Barber<br>◯5 Zaouia<br>◯9 Dont know	

17. GYNECO-OBSTETRICAL HISTORY (WOMEN)			
**No**	**Questions**	**Answers**	**Skip**
**Q110**	How many times have you given birth?	|__||__| *(Number of times (0 if never)*<br>If never	Q114
**Q111**	During delivery, did you have:<br>Vacuum<br>forceps<br>C section<br>Episiotomy<br>Normal delivery	*(Nombre de fois, si jamais 0)*<br>|__| |__| |__| |__| |__|	
**Q112**	Your delivery took place in the presence of<br>Doctor<br>Nurse<br>Midwife<br>Other (specify)	*(Number of times (0 if never)*<br>|__| |__| |__| ---	
**Q113**	Where did you give birth?<br>Hospital<br>Health center<br>Private clinic<br>At home<br>Other (specify)	*(Number of times (0 if never)*<br>|__| |__| |__| |__| ---	
**Q114**	Have you had spontaneous miscarriages?	|__||__| *(Number of times (0 if never)*<br>If never	Q116
**Q115**	Where did the miscarriage occur?<br>Hospital<br>Health center<br>Private clinic<br>At home<br>Other (specify)	*(Number of times (0 if never)*<br>|__| |__| |__| |__| ---	
**Q116**	Have you had induced abortions?	|__| *(Number of times (0 if never)*<br>*If never*	Q118
**Q117**	Where did the induced abortion take place?<br>Hospital<br>Health center<br>Private clinic<br>At home<br>Other (specify)	*(Number of times (0 if never)*<br>|__| |__| |__| |__| ---	
**Q118**	Have you used any of the following contraceptive methods?<br>IUD<br>Injectables<br>Implants (1 Yes, 0 No)	*(1 Yes, 0 No)*<br>|__| |__| |__|	
**Q119**	Have you had any gynecological surgery? If yes, specify the type	|__| *(1 Yes, 0 No)*<br>---	

We thank you for your time and attention.

Blood sample taken ◯ *0 No,1 Yes*

Date of blood sampling |__|__|__|__|__|__|__|__|
D D M M Y Y Y Y

	Name and Surname	Signature	Date	Notes
Interviewer				
Supervisor				
Data Entry				

TEST RESULTS			
**Q120**	Total anti-HBc antibodies	|__| (0 negative, 1 positive, 9 Not Done)	
**Q121**	HBs Antigen (HBsAg)	|__| (0 negative, 1 positive, 9 Not Done)	
**Q122**	Anti-HBs antibodies	|__| (0 negative, 1 positive, 9 Not Done)	
**Q123**	Anti-HCV antibodies	|__| (0 negative, 1 positive, 9 Not Done)	
