# Supplementary material for: National Prevalence and Risk Factors of Hepatitis B Virus Infection in Tunisia Two Decades After Vaccine Introduction
Source: Vaccines (Basel). 2026 Apr 23;14(5):373. doi: 10.3390/vaccines14050373 (PMC13211386; doi:10.3390/vaccines14050373)
Supplement: Supplementary file 1 [file vaccines-14-00373-s001.zip › 20141215_National_Survey_Prevalence of VHB_Tunisia_Minor_Survey_English_Version.pdf]

Entry Number |_|_|_|_|_|

ANONYMOUS DATA COLLECTION FORM
INFANTS AND ADOLESCENTS RECORDS

PARTICIPANTS LESS THAN 18 YEARS

INDIVIDUAL Identification

Unique Identifier: (UI) :

|_|_|_| |_|_|_| |_|_|_| |_|_| |_|_|_|

Delegation code   Family code   N° of individual

Name and surname of the individual :

PART ONE: SOCIO-DEMOGRAPHIC DATA

1. IDENTIFICATION DU SUJET			
**N°**	**Questions**	**Answers**	**Skip**
Q1	Gender	◯1 male 2 Female	
Q2	Date of birth<br>(If unknown, specify age)	|_|_|_|_|_|_|_|_| D D M M Y Y Y Y<br>|_|_|_| years	
Q3	Date of survey	|_|_|_|_|_|_|_|_| D D M M Y Y Y Y	
Q4	Gouvernorate of residence	|_|_|	
Q4	Gouvernorate of residence	|_|_|_|	
Q5	Exact Address<br>N° Phone number	---<br>---<br>---	
Q6	Have you lived here all your life?	◯ (1 Yes, 0 No)<br>if No	Q8
Q7	If No, how long have you lived here?<br>(Don't know / no response code 999)	|_|_|_| Years	
**2. EDUCATION AND OCCUPATION**			
Q8	What is your current level of education?	◯ 0 Never attended school<br>◯ 1 Kotteb, preschool<br>◯ 2 Primary<br>◯ 3 Middle school<br>◯ 4 High school<br>◯ 9 Below the age	
Q9	Do you currently have a professional activity?	◯ (1 Yes, 0 No)<br>If yes, specify -----------------------------	
**3. CIRCUMCISION (BOYS ONLY)**			
Q10	At what age were you circumcised?	|_|_|ans	
Q11	Who performed your circumcision?	◯1 Doctor<br>◯2 Nurse<br>◯ Traditional circumciser,<br>◯ 9 Don't know	
Q12	Where was your circumcision performed?	◯ 1 At home<br>◯ 2 Hospital<br>◯ 3 Private<br>◯ clinic,<br>◯ 4 Barber<br>◯ 5 Zaouia<br>9 Dont know	

4. MEDICAL AND SURGICAL HISTORY			
**N°**	**Questions**	**Answers**	**Skip**
**Q13**	Are you being treated for any disease? If Yes, specify up to 3 chronic illnesses	◯ (1 Yes, 0 No, 9 Don't know)<br>Specify 1 ---------------------------|___|__| Y|___|__| M<br>Specify 2---------------------------|___|__| Y |___|__| M<br>Specify 3 ---------------------------|___|__| Y |___|__| M	
**Q14**	Are you hemophilic? If Yes, since when?	◯ (1 Yes, 0 No, 9 Don't know)<br>|___|__| year |___|__| month	
**Q15**	Are you on chronic dialysis?	◯ (1 Yes, 0 No, 9 Don't know)<br>|___|__| year |___|__| month	
**Q16**	Have you had an organ transplant?	◯ (1 Yes, 0 No, 9 Don't know)<br>|___|__| year |___|__| month	
**Q17**	Have you had surgery? If Yes, specify type and since when	◯ (*0 No, 1 Once, 2 More than once*) ***If No***<br>Type 1 ---------------------------|___|__| Y|___|__| M<br>Type 2 ---------------------------|___|__| Y|___|__| M<br>Type 3 ---------------------------|___|__| Y|___|__| M	Q19
**Q18**	Where were your surgery/s performed?<br>Hospital<br>Private clinic<br>Other	(*1 Once, 2 More than once*)<br>|__| Specify ---------------------------------<br>|__| Specify ---------------------------------<br>|__| Specify ---------------------------------	
**Q19**	Have you ever had an injection before?	◯ (1 Yes, 0 No, 9 Don't know) If ***No***	Q24
**Q20**	If Yes, how many injections in your life?	|_| Less than 10<br>|_| More than 10<br>|_| Regularly<br>|_| Don't know	
**Q21**	Usually, who administers your injections?<br><br>Doctor / Nurse / Pharmacist /<br>Other<br>health personnel /<br>Barber /<br>Other (Specify)	(*0 No, 1 Occasionally, 2 Frequently, 3 Always, 9 Don't know*)<br>|__| |__| |__| |__| |__| ---	
**Q22**	Injections usually given with:<br>Open plastic syringe in front of you<br>Closed plastic syringe<br>Glass syringe	(*1 Yes, 0 No, 9 Don't know*)<br>|__| |__| |__|	
**Q23**	Type of injection:<br>injection SC<br>injection IM<br>injection IV	(*1 Yes, 0 No, 9 Don't know*)<br>|__| |__| |__|	
**Q24**	Have you been hospitalized? If Yes, specify hospital/clinic	◯ (1 Yes , 0 No) ***if no***<br>*specify* --	Q29
**Q25**	How many times?	|___|__|	
**Q26**	Year(s) of hospitalization	|__|__|__|__| |__|__|__|__| |__|__|__|__|	

5. SURGICAL AND HOSPITAL PROCEDURES			
**N°**	**Questions**	**Answers**	**Skip**
**Q27**	Reason for hospitalization<br>Diagnosis & Treatment<br>Surgery<br>Pregnancy & Delivery<br>Other (Specify)	*(0 No, 1 Once, 2 More than once)*<br>|__| |__| |__| |__|--	
**Q28**	During hospitalization, did you have any of the following procedures?<br>Biopsy<br>Urinary catheterization<br>Blood catheterization<br>Hemodialysis<br>Coronary angiography<br>Upper digestive endoscopy<br>Lower digestive endoscopy<br>Bronchoscopy<br>Laparoscopy<br>Arthroscopy<br>Hysteroscopy (Women)<br>Sutures<br>Invasive radiology exams<br>Mesotherapy<br>Acupuncture<br>Varicose vein sclerotherapy<br>Other (Specify)	*(Nb of times, 0 Never)*<br>|__| |__| |__| |__| |__| |__| |__| |__| |__| |__| |__| |__| |__| |__| |__| |__| |__|--	
6. DENTAL TREATMENT			
**Q29**	Have you received dental care?	◯ (1 Yes , 0 No) if no	Q32
**Q30**	If yes, specify :<br>Gum treatment<br>Scaling<br>Tooth extraction<br>Local anesthesia for extraction<br>Other (Specify)	*(0 No, 1 Once, 2 More than once)*<br>|__| |__| |__| |__| |__|--	
**Q31**	Usual place for dental care<br>Hospital dentist<br>Private dentist<br>Barber<br>Other Specify))	*(0 No, 1 Once, 2 More than once)*<br>|__| |__| |__| |__|--	
7. TATTOO, SCARRING, EAR PIERCING, ACUPUNCTURE			
**Q32**	Have you ever had a tattoo?	|__||__| *(Number of times (0 if never)*	
**Q33**	Have you ever had a scarification?	|__||__| *(Number of times (0 if never)*	
**Q34**	Have you ever had acupuncture ?	|__||__| *(Number of times (0 if never)*	
**Q35**	Have you ever had a piercing?	|__||__| (1 Yes , 0 No)	

8. HEPATITIS B HISTORY			
**Q36**	Have you been vaccinated against hepatitis B?	|__| (1 Yes, 0 No, 9 Don't know) if No	Q38
**Q37**	If Yes, how many doses?<br>dates:<br>Dose 1<br>Dose 2<br>Dose 3	|__| <br>|__|__|__|__| |__|__|__|__| |__|__|__|__|	
**Q38**	Have you ever had acute hepatitis B diagnosed by a physician?	|__| (1 Yes, 0 No, 9 Don't know) if No	Q40
**Q39**	If Yes, was HBs antigen still positive after infection?	|__| (1 Yes, 0 No, 9 Don't know)	
**Q40**	Do you know if a close relative is a chronic carrier of hepatitis B (HBs antigen)?	|__| (1 Yes, 0 No, 9 Don't know) if No	Q42
**Q41**	If Yes, what is their relationship to you	○ 1 Father<br>○ 2 Mother<br>○ 3 Brother<br>○ 4 Sister<br>○ 9 Other, specify	
**Q42**	Have you ever had a blood test for HBs antigen? If yes, specify context	|__| (1 Yes, 0 No, 9 Don't know) if No	
**Q43**	During routine health check	|__| (1 Yes, 0 No, 9 Don't know)	
**Q44**	Before hepatitis B vaccination	|__| (1 Yes, 0 No, 9 Don't know)	
**Q45**	Due to a risk factor	|__| (1 Yes, 0 No, 9 Don't know)	
**Q46**	Due to blood test showing liver anomaly (transaminases)	|__| (1 Yes, 0 No, 9 Don't know)	
**Q47**	During treatment of liver disease	|__| (1 Yes, 0 No, 9 Don't know)	
**Q48**	Other (Specify)--		
**Q49**	Have you ever had a negative result for this/these test(s)?	|__| (1 Yes, 0 No, 9 Don't know) if No	Q51
**Q50**	If Yes, date of last negative result	|__||__|month |__||__| Year	
**Q51**	Have you ever had a positive result for this/these test(s)?	|__| (1 Yes, 0 No, 9 Don't know) if No	Q53
**Q52**	If Yes, date of last positive result	|__||__|month |__||__| Year	
**Q53**	Are/were you followed by a physician for this infection?	|__| (1 Yes, 0 No) if No	Q57
**Q54**	If Yes, type of physician	1 General Practitioner, 2 Specialist, 3 Both	
**Q55**	If followed by a physician, location?	○1 In town, 2 Hospital	
**Q56**	Have you ever been told you had hepatitis B in the past and were cured?	|__| (1 Yes, 0 No, 9 Don't know)	

9. HEPATITIS C HISTORY			
**Q57**	Have you ever had hepatitis C serology (anti-HCV)? Specify context	|__| (1 Yes, 0 No, 9 Don't know)	Q73
**Q58**	During routine health check	|__| (1 Yes, 0 No, 9 Don't know)	
**Q59**	During blood donation	|__| (1 Yes, 0 No, 9 Don't know)	
**Q60**	Due to a risk factor	|_| (1 Yes, 0 No, 9 Don't know)	
**Q61**	During blood test showing liver anomaly	|_| (1 Yes, 0 No, 9 Don't know)	
**Q62**	During liver disease treatment	|_| (1 Yes, 0 No, 9 Don't know)	
**Q63**	Due to partner's known positive HCV serology	|_| (1 Yes, 0 No, 9 Don't know)	
**Q64**	Due to family member's known positive HCV serology	|_| (1 Yes, 0 No, 9 Don't know)	
**Q65**	Other (Specify) --		
**Q66**	Have you ever had a negative result for this/these test(s)?	|_| (1 Yes, 0 No, 9 Don't know)	Q68
**Q67**	If Yes, date of last negative result	|__||__|month |__||__| Year	
**Q68**	Have you ever had a positive result for this/these test(s)?	|__| (1 Yes, 0 No, 9 Don't know)	Q70
**Q69**	If Yes, date of last positive result	|__||__|month |__||__| Year	
**Q70**	Are/were you followed by a physician for this infection	|_| (1 Yes, 0 No) if No	Q73
**Q71**	If Yes, type of physician	1 General Practitioner, 2 Specialist, 3 Both	
**Q72**	If followed by a physician, location	○1 In town, 2 Hospital,	
**Q73**	Do you know if a close relative had positive HCV serology?	|__| (1 Yes, 0 No, 9 Don't know)	Q77
**Q74**	If Yes, relationship to you	○ 1 Father<br>○ 2 Mother<br>○ 3 Brother<br>○ 4 Sister<br>○ 9 Other (Specify)	

We thank you for your time and attention.

Blood Sample Taken ◯ (0 No,1 Yes)

Date of blood sampling |__|__|__|__|__|__|__|__|
D D M M Y Y Y Y

	Name and Surname	Signature	Date	Notes
Interviewer				
Nurse (Blood collector)				
Supervisor				
Data Entry				

TEST RESULTS			
Q75	Total anti-HBc antibodies	|__| (0 negative, 1 positive, 9 Not Done)	
Q76	HBs Antigen (HBsAg)	|__| (0 negative, 1 positive, 9 Not Done)	
Q77	Anti-HBs antibodies	|__| (0 negative, 1 positive, 9 Not Done)	
Q78	Anti-HCV antibodies	|__| (0 negative, 1 positive, 9 Not Done)	
